# An Adaptive Weighted KNN Positioning Method Based on Omnidirectional Fingerprint Database and Twice Affinity Propagation Clustering

**DOI:** 10.3390/s18082502

**Published:** 2018-08-01

**Authors:** Jingxue Bi, Yunjia Wang, Xin Li, Hongxia Qi, Hongji Cao, Shenglei Xu

**Affiliations:** NASG Key Laboratory of Land Environment and Disaster Monitoring, China University of Mining and Technology, Xuzhou 221116, China; bjx1050@163.com (J.B.); linuxcumt@126.com (X.L.); hongxiaqi@yeah.net (H.Q.); hjcao@cumt.edu.cn (H.C.); cumtxsl@163.com (S.X.)

**Keywords:** indoor positioning, adaptive weighted algorithm, K-nearest neighbor, omnidirectional fingerprint database, affinity propagation clustering

## Abstract

The human body has a great influence on Wi-Fi signal power. A fixed K value leads to localization errors for the K-nearest neighbor (KNN) algorithm. To address these problems, we present an adaptive weighted KNN positioning method based on an omnidirectional fingerprint database (ODFD) and twice affinity propagation clustering. Firstly, an OFPD is proposed to alleviate body’s sheltering impact on signal, which includes position, orientation and the sequence of mean received signal strength (RSS) at each reference point (RP). Secondly, affinity propagation clustering (APC) algorithm is introduced on the offline stage based on the fusion of signal-domain distance and position-domain distance. Finally, adaptive weighted KNN algorithm based on APC is proposed for estimating user’s position during online stage. K initial RPs can be obtained by KNN, then they are clustered by APC algorithm based on their position-domain distances. The most probable sub-cluster is reserved by the comparison of RPs’ number and signal-domain distance between sub-cluster center and the online RSS readings. The weighted average coordinates in the remaining sub-cluster can be estimated. We have implemented the proposed method with the mean error of 2.2 m, the root mean square error of 1.5 m. Experimental results show that our proposed method outperforms traditional fingerprinting methods.

## 1. Introduction

The indoor positioning technology is a research focus on navigation and location-based services (LBS), and has attracted extensive attentions of research institutions, universities and enterprises. Nowadays, there are a large number of applications for ILBS in market. And researchers have carried out a large number of studies on indoor positioning technologies and developed corresponding indoor positioning systems, such as the infrared [1], ultrasonic [2] or sound [3], radio frequency identity [4,5] (RFID), ZigBee [6], Wi-Fi [7,8], Bluetooth [9], microelectromechanical systems (MEMS) sensors [10], ultra-wideband [11] (UWB), geomagnetic field [12,13], visible light [14], computer vision [15], pseudolites [16] (PL), etc. However, due to the complexity of the indoor environment, it is usually difficult to provide a satisfactory level of accuracy in most applications. Among them, the Wi-Fi fingerprinting technology is the most feasible and cost effective technique for indoor localization, without knowing locations of APs in advance.

The fingerprinting localization contains two stages, offline stage and online stage. The basic idea of fingerprinting localization for estimating user’s position is matching the online received signal strength (RSS) readings with offline prebuilt fingerprint database. The fingerprint database, also named as radio map, consists of a large number of reference points (RPs), where RSS measurements are collected with known positions. In general, there are two fingerprinting localization approaches. One effective solution uses the K-nearest neighbor algorithm (KNN) to estimate users’ position by computing the centroid of the K closest neighbors. It is easy to implement but offers poor accuracy. The other approach is statistical analysis based on the probabilities of each RP calculated by Bayesian theory [17] or kernel functions [18]. This approach has high computational complexity, which makes it difficult to run on mobile devices with poor processing capability and short battery life.

To improve positioning accuracy and reduce computational cost, clustering approaches have been introduced in fingerprinting localization. In the offline stage, a fingerprint database is divided into several clusters. In the online stage, the online RSS readings are used for cluster matching to determine which cluster the online RSS readings belong to, and then similarity is calculated between the online RSS readings and RP only in the selected cluster rather than the whole fingerprint database. Obviously, this is an effective way to avoid large localization errors and reduce the burden on computation. Thus, many researchers have proposed many clustering-based fingerprinting approaches, such as K-means [19], fuzzy c-means [20] (FCM), affinity propagation clustering [21] (APC), and support vector machine clustering [22]. The above clustering approaches took the signal-domain distance as the clustering feature, causing a not actual location distribution of RPs, Li [23] conducted clustering based on the fusion of the signal-domain and position-domain distances with good clustering performance. Due to continuous radio propagation, unpredictable radio channel attenuation, signal shadowing, multipath interference, and even dynamic indoor environment, there are no absolutely separated cluster margins for fingerprints. Transition region should exist between adjacent clusters, so it is not suitable for fingerprinting localization based on SVM clustering algorithm [22], which focuses on the margin between two canonical hyperplanes.

To cope with these dynamical environment changes, researchers have proposed several kinds of methods. Inertial sensor-based methods make use of human trajectories to rapidly construct fingerprint database, such as Zee [23] and LiFS [24]. Alshami [25] utilizes a path-loss model to generate RSS fingerprints considering the attenuation of walls, floors, and human body. Luo [26] employs a self-locating mobile robot to continuously collect RSS measurement for autonomously updating fingerprint database. Eisa [27] builds a lightweight and robust radio map by filtering unless APs and fingerprints. They pay more attention to fingerprint database automatic construction and update. For adaptive positioning algorithms, more researchers are likely to focus on the signal model-based method [28] to dynamically respond to changes in the environment, instead of fingerprinting methods. With respect to weighted calculation process, Li [29] has found that when K is 5, a good positioning performance is obtain by KNN or weighted KNN (WKNN). In fact, indoor scene is a complex, dynamic and changing environment. The fixed value of K may decrease the positioning accuracy. Shin [30] has proposed an enhanced weighted KNN (EWKNN) technique to filtrate several RPs for calculating weighted coordinates, but it ignored the real spatial distribution of RPs and may lead to larger localization errors.

This study proposes a localization approach based on the omnidirectional fingerprint database (OFPD) and twice APC algorithm. The method also consists of two stages. On the offline stage, we first build an OFPD, which is the table of RSS measurements, orientations and positions. After building OFPD, all RPs are clustered by APC algorithm, and two adjacent clusters are adjusted based on their transition region. The online stage comprises two steps: cluster matching, selecting the cluster to which the online RSS readings belong, and the proposed adaptive weighted KNN (AWKNN) algorithm. In the latter step, the online RSS readings are matched with the selected cluster for obtaining K initial RPs with the smallest signal-domain distances by KNN. After clustering for the K selected initial RPs by APC, the most probable sub-cluster with several RPs is reserved, and the user’s position can be estimated by inverse distance weighted algorithm based on all RPs’ corresponding signal-domain distances in the reserved sub-cluster.

The remainder is organized as follows: Section 2 describes the proposed two-stage localization approach based on OFPD and twice affinity propagation clustering. The experimental testbed is introduced and the performance of the proposed method is evaluated in Section 3. Section 4 discusses the results and limitations. We conclude the work in Section 5.

## 2. The Proposed Indoor Localization Method

### 2.1. Overall Structure of the Proposed Indoor Localization Method

**Figure 1:**
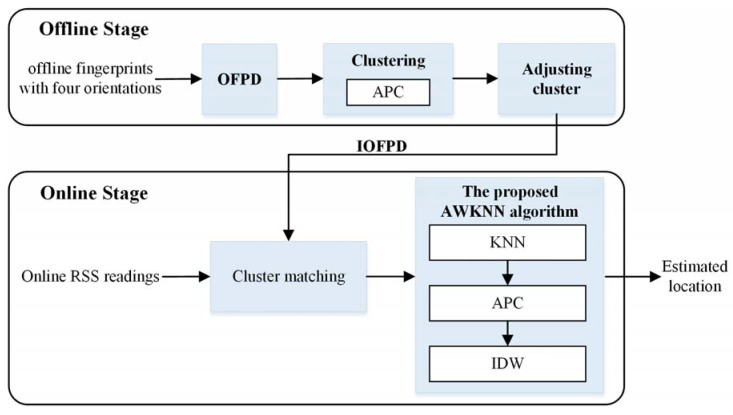
Flow chart of the proposed indoor localization method.

The main aim of the proposed indoor localization method is to estimate the user’s current position with good positioning performance by the online RSS readings on a Wi-Fi integrated mobile device. As shown in Figure 1, the proposed approach consists of two stages: the offline stage and online stage. On the offline stage, RSS fingerprints are collected at specified locations with four orientations in testing area, and the OFPD is built by the combination of collected RSS fingerprints, locations and orientations; then clustering are conducted to divide all RPs of OFPD into several clusters by APC; subsequently, the improved omnidirectional fingerprint database (IOFPD) can be obtained after adjusting cluster based on transition region. The offline clustering scheme is processed based on the fusion of signal-domain distance and position-domain distance between each RP. This process can ensure that the clustering results are consistent with actual indoor scenes and structures. The user’s real-time location can be estimated on the online stage by matching the online RSS readings with the IOFPD. The online stage is composed of cluster matching and the proposed AWKNN algorithm. Cluster matching is used to select the most appropriate cluster in the IOFPD for the further calculation. The AWKNN algorithm contains three parts: KNN, APC and IDW. KNN algorithm is used for selecting K initial RPs with top smallest signal-domain distances from the selected cluster by cluster matching. APC algorithm divides these K RPs into several clusters. The online APC is conducted based on position-domain distance between the K RPs. It is the difference between the offline APC and online APC. Then, the optimal cluster is selected for estimating location by IDW algorithm.

As far as we know, cluster matching or cluster recognition is a challenging task, and the accuracy of cluster recognition is usually poor with a single means. While a fusion method with MEMS sensors or camera has a good performance. This paper focuses on Wi-Fi fingerprinting method for indoor localization, thus cluster matching with multi-sensor fusion is no longer introduced in detail.

In addition, KNN algorithm is the simplest one of machine learning methods. And KNN is firstly introduced for Wi-Fi fingerprinting localization in [7] in detail. Thus, the algorithm in regard to KNN will not be described in the following section.

### 2.2. The Omnidirectional Fingerprint Database (OFPD)

The traditional fingerprinting localization methods usually utilize the common fingerprint database (CFPD), which taking the average of RSS measurements collected at RPs from hearable APs in different orientations or single direction [7]. The maps of MAC address and corresponding RSS mean value are named as RSS fingerprints, expressed as:

(1) $$R_{i}=\left[\begin{array}{c} \begin{array}{cc} MAC_{i,1} & \bar{RSS_{i,1}} \end{array}\\ \begin{array}{cc} MAC_{i,2} & \bar{RSS_{i,2}} \end{array}\\ \cdots \\ \begin{array}{cc} MAC_{i,m} & \bar{RSS_{i,m}} \end{array} \end{array}\right]\;$$

Our proposed OFPD is a table, including RSS fingerprints, corresponding orientations and positions. It stores RSS fingerprints at known locations in single direction. The orientation may be one of the front and back, right and left along the building. As shown in Table 1, **id** refers to the record number, **pid** refers to the point number of RP, ***x*** and ***y*** refer to the coordinate values of RP, **MAC** refers to the MAC address of AP, and **RSS** refers to corresponding RSS value in the unit of dBm. The RSS fingerprint is the average of RSS measurements collected at one RP in one orientation. In other words, there are four PRs at the same location in the OFPD. Moreover, the aim of building an OFPD is to get more adjacent neighbors in the online stage for fine localization.

### 2.3. Affinity Propagation Clustering

#### 2.3.1. The Similarity Based on Signal-Domain and Position-Domain Distances

Unlike traditional clustering-based fingerprinting methods, our clustering during offline stage is conducted based on the similarity, the normalized hybrid distance, which is the fusion of the signal-domain distance and position-domain distance. The advantage of taking the hybrid distance as the similarity is that the clustering results can reflect the exact location relationship of RPs.

The signal-domain distance can be calculated by:

(2) $$d_{sig}\left(l_{i},l_{j}\right)={\left\|R_{i},R_{j}\right\|}^{p}={\left(\sum_{k=1}^{m}{\left(RSS_{i,k}-RSS_{j,k}\right)}^{p}\right)}^{1/p}\;$$

where *d_sig_*(*l_i_*, *l_j_*) denotes the signal-domain distance, **R***_i_* denotes the RSS fingerprint at *l_i_* (the *i*th location), *RSS_i,k_* denotes the RSS value of the *k*th AP and *m* is the amount of same APs, and the signal-domain distance can be Manhattan distance and Euclidean distance when the superscript *p* is 1 and 2, respectively. In our analysis, we use the latter to measure the similarity between two sample points in both signal space and position space.

However, it is difficult for the user’s mobile device to detect the fixed *m* same APs at every location in a building. In other words, the number of detected same APs is dynamically changing. The similarity measurement with fewer same APs may be smaller located at a faraway place, while the similarity measurement with more same APs may be bigger at a near location. Thus, to compare similarity measurements fairly, the average signal-domain distance is proposed by introducing the amount of same detected APs in Equation (3):

(3) $$d_{sig-avg}\left(l_{i},l_{j}\right)=\sqrt{\sum_{k=1}^{m}{\left(RSS_{i,k}-RSS_{j,k}\right)}^{2}/m}\;$$

The position-domain distance can be calculated by Equation (4):

(4) $$d_{pos}\left(l_{i},l_{j}\right)=\sqrt{{\left(x_{i}-x_{j}\right)}^{2}+{\left(y_{i}-y_{j}\right)}^{2}}\;$$

Due to different metrics of signal-domain distance and position-domain distance, the normalization should be introduced for further process. The normalized hybrid distance could be obtained by Equation (5), where *Nor*() is the normalized function. In this study, the *min-max* normalization method is introduced:

(5) $$d_{hyb}\left(l_{i},l_{j}\right)=Nor\left(d_{sig-avg}\left(l_{i},l_{j}\right)\right)\times Nor\left(d_{pos}\left(l_{i},l_{j}\right)\right)\;$$

#### 2.3.2. Clustering by Affinity Propagation Algorithm

In the usual clustering algorithms, cluster centers are iteratively chosen and optimized to minimize the quadratic sum of the distance between the cluster center and other members of the cluster. The classic K-means algorithm and fuzzy C-means algorithms should be provided a series of initial cluster centers and assigned K or C value (the specified number of clusters) when starting. Instead, APC algorithm automatically creates clusters based on the constant exchanging of reading similarities between the RPs without specifying the number of clusters [31].

We use the similarity *s*(*i*, *j*) to describe how well the *RP_j_* at location *l_j_* is suited to be the cluster center for the *RP_i_*. Instead of requiring the number of clusters, affinity propagation takes as input a real number *s*(*j*, *j*) for each point *RP_j_*. The larger the value *s*(*j*, *j*) is, the more likely the point *RP_j_* is chosen as cluster center. These values of *s*(*j*, *j*) are referred as “preferences”. The number of clusters is influenced by the values of preferences. The preferences are set to a common value, which leads to produce different numbers of clusters. If the value is set as the median of the input similarities, algorithm will produce a moderate number of clusters. If the value is the minimum similarities, a small number of clusters will be obtained.

The similarity *s*(*i*, *j*) can be calculated by Equations (6) or (7). Each similarity is set to a negative value. On the offline stage, it is a negative normalized hybrid distance in Equation (6). While on the online stage, it is set to a negative squared Euclidean distance in position domain in Equation (7). Where *i*, *j* denote the index of RP, respectively:

(6) $$s\left(i,j\right)=-d_{hyb}\left(l_{i},l_{j}\right)\;$$

(7) $$s\left(i,j\right)=-{\left\|l_{i}-l_{j}\right\|}^{2}=-\left({\left(x_{i}-x_{j}\right)}^{2}+{\left(y_{i}-y_{j}\right)}^{2}\right),\forall i,j\in \left\{1,2,\ldots ,N\right\}\;$$

There are two kinds of message exchanged between all RPs: responsibility and availability. Two kinds of messages can be combined to determine which points are cluster center and, for every other point, which cluster center it belongs to:

- the responsibility *r*(*i*, *j*): sent from the *RP_i_* to candidate cluster center *RP_j_*, which reflects the accumulated evidence for how well-suited the *RP_j_* is to serve as the cluster center for the *RP_i_*;
- the availability *a*(*i*, *j*): sent from the candidate cluster center *RP_j_* to the *RP_i_*, which reflects the accumulated evidence for how appropriate it would be for the *RP_i_* to choose the *RP_j_* as its cluster center.

The responsibilities can be computed by Equation (8). To begin with, the availabilities are initialized to zero: *a*(*i*, *j*) = 0:

(8) $$r\left(i,j\right)=s\left(i,j\right)-\underset{j^{\prime }\neq j}{\max}\left\{a\left(i,j^{\prime }\right)+s\left(i,j^{\prime }\right)\right\}\;$$

In the first iteration, *RP_j_* is set as cluster center, because the availabilities are zero, *r*(*i*, *j*) is set to the input similarity between *RP_i_* and *RP_j_* (cluster center) minus the largest similarity between *RP_i_* and other candidate cluster center *RP_j′_*. In later iterations, when some points are assigned to other cluster centers, the availabilities will drop below zero, decreasing the effectiveness of some of the input similarities *s*(*i*, *j*′).

When *j* = *i*, the responsibility *r*(*j*, *j*) is set to the input preference *s*(*j*, *j*) minus the largest similarity between *RP_i_* and other candidate cluster center *RP_j′_*. This “self-responsibility” reflects accumulated evidence that *RP_j_* is set as a cluster center. It can be computed by Equation (9). In our procedure, during both the offline and online stage, the preference is set as the median of the input similarities to acquire a moderate number of clusters:

(9) $$r\left(j,j\right)=s\left(j,j\right)-\max\left\{s\left(i,j^{\prime }\right)\right\}\;$$

The following availability update gathers evidence from points *RP_i_* to the candidate cluster center *RP_j_* whether it could play a good role of cluster center. The availability *a*(*i*, *j*) is set to the self-responsibility *r*(*j*, *j*) plus the sum of the positive responsibilities, which denotes the candidate cluster center receives from other points *RP_i′_*. It can be obtained by Equation (9). The availability *a*(*i*, *j*) as a cluster center can be increased if some other points *RP_i’_* have positive responsibilities for point *RP_j_*:

(10) $$a\left(i,j\right)=\min\left\{0,r\left(j,j\right)+\sum_{i^{\prime }\neq i,j}\max\left\{0,r\left(i^{\prime },j\right)\right\}\right\}\;$$

To limit the influence of strong incoming positive responsibilities, the total sum is set as threshold value. So that the availability will not go above zero. The “self-availability” *a*(*j*, *j*) can be updated by Equation (11), which reflects accumulated evidence that the *RP_j_* as a cluster center sent to the candidate center from other RPs:

(11) $$a\left(j,j\right)=\sum_{i^{\prime }\neq j}\max\left\{0,r\left(i^{\prime },j\right)\right\}\;$$

So far, two kinds of messages can be computed and exchanged between pairs of RPs, and the procedure can run from Equations (8) to (11) with known similarities. During affinity propagation, responsibilities and availabilities are combined to identify cluster center. For point *RP_i_*, the point *RP_j_* that maximizes *r*(*i*, *j*) + *a*(*i*, *j*) either identifies *RP_i_* as a cluster center if *j* = *i*, or identifies the point *RP_j_* that is the cluster center for *RP_i_*.

The message-passing procedure may be terminated after a fixed number of iterations, after changes in the messages fall below a threshold, or after cluster center stay constant. In our procedure, these three termination decisions are combined for terminating procedure. We set the number of iterations is 1000. The message threshold can be obtained by Equation (11). The distance between the last cluster center and the new one is extremely small. Each of conditions meets the requirements, the procedure will terminate.

The iteration of affinity propagation can be concluded into three steps:

- updating all responsibilities given the availabilities,
- updating all availabilities given the responsibilities,
- combining responsibilities and availabilities to determine when the algorithm should be terminated.

### 2.4. Adjusting Cluster Based on Transition Region

**Figure 2:**
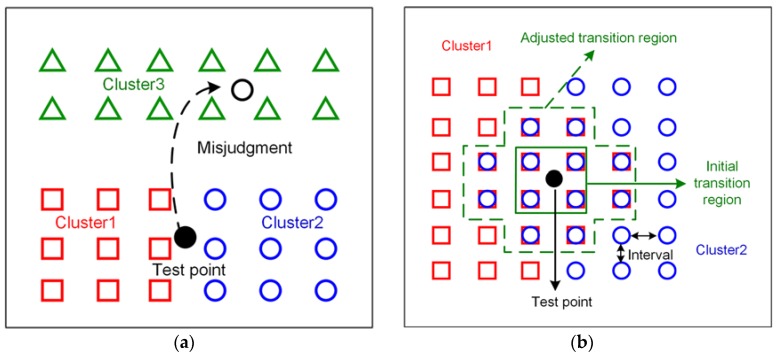
The phenomena of cluster misrecognition and the illustration of adjusting cluster based on transition region, (**a**) misjudgments usually take place on the margin between two adjacent clusters and dynamic huge RSS readings change; (**b**) the marked area in dash box is the adjusted transition region, it belongs to both the Cluster 1 and the Cluster 2.

During offline stage, we measured RSS fingerprints heard from surrounding APs at the same locations in four orientations. After clustering by APC based on the normalized hybrid distance, we can find that an area in solid box in Figure 2b. RPs in this solid box belong to both Cluster 1 and Cluster 2. This overlapping area is called the transition region for convenience. 

Wireless signals are easily influenced by unpredictable channel attenuation, signal shadowing, multipath interference and dynamic indoor environment, which make RSS distributions different surrounding the human body, causing overlapping areas. However, almost all clustering results for fingerprinting localization in other studies are completely separate. These results may be not practical for RPs evenly distributed with small intervals in a testing area, leading to misjudgments when the test points are located at the transition region. Moreover, because of the endlessly dynamic changing RSS readings, it might be identified to belong to another cluster by mistake. If the mobile device locates at this transition region and the employed radio map is without transition region, no matter which cluster the mobile device belongs to, the large localization error may be introduced into cluster matching or the following localization. Therefore, transition region is factual and critical for the online stage.

Assuming that the online RSS readings are identified to belong to Cluster 1 by cluster matching with the radio map, it includes the transition region, and the real position is in transition region. K RPs in Cluster 1 will be selected by KNN based on similarities between this RSS readings and all RPs in Cluster 1. Then the selected K RPs are used for calculating coordinates by weighted mean method. How many selected RPs belong to the transition region for location estimation? The number is uncertain because of the dynamic and changeable wireless environment. There is no doubt that the more RPs are selected in this transition region, the smaller the localization errors are. Enlarging the transition region can ensure that less distant RPs will be selected for location estimation. Thus, enlarging the transition region contributes to avoid large localization errors. For two adjacent clusters, no matter which cluster the test point is identified to belong to, the enlarging transition region means that more RPs in this area are likely selected for estimating location without large localization error. Therefore, adjusting clusters by extending transition regions can alleviate the influence of cluster misjudgment and avoid large localization errors to some extent.

Figure 2 shows the sketch map of cluster misjudgment and adjusting cluster based on transition region. In order to explain it clearly, we assume that the employed radio map does not include transition region. In Figure 2a, three clusters are illustrated in different colors and various shapes. From this figure, we can see that the Cluster 1 and Cluster 2 are adjacent clusters, and the Cluster 3 is the separated one far away from them, the test point is located at the area of margins between two adjacent clusters. It is obvious that it is a mistake no matter which cluster the test point belongs to. And even supposing that the test point is assigned to belong to the Cluster 1 by cluster matching on the online stage. Several RPs will be selected to estimate position, while these selected RPs are actually apart from the test point in position space, causing a large positioning error. In addition, because of the dynamic RSS readings, the test point may be determined to belong to the Cluster 3 by mistake. This misjudgment can be avoided by the fusion of multi-sensor, such as accelerometer, gyroscope and geomagnetic filed sensor, or under the constraint of pedestrian distance, so it will not be described in this paper. Our focus is on cluster misjudgment caused by adjacent clusters. As the above description on transition region, it is factual at the margin of two adjacent clusters. And extending the coverage of transition region can alleviate cluster misjudgment. Figure 2b illustrates the algorithm to adjust clusters based on transition region. The selected area in solid box is initial transition region of two adjacent clusters after clustering in the OFPD. RPs in two adjacent clusters are with same positions. They can be obtained by traversing both two clusters. We select RPs in the range of interval, which is the sample distance between RPs during offline stage, denoted as *d_int_* in Algorithm 1. The value is a constant. The adjusted transition region, marked in dash box in Figure 2b, is combined with the new selected RPs and RPs in initial transition region. The range of transition region is extended from the solid box to the dash box. The new selected RPs are added into each adjacent clusters. Each cluster center should be updated based on the new adjusted cluster. At last, all clusters and their centers are update. The IOFPD are made up of these adjusted clusters and used for the following online stage. The detailed descriptions of algorithm to adjust clusters is shown as follows.

**Algorithm 1:** The algorithm to adjust clusters based on transition region1. **Input:** OFPD after clustering by affinity propagation2. **Output:** IOFPD3. *N_cls_* = Num(*C*), *C* denotes clusters in OFPD, *d_int_* is a fixed constant value;4. **for**
*j* = 1 to *N_cls_* − 1 **do**5.  *N_1_* = Num(*C_j_*), *N_2_* = Num(*C_j+1_*), *C_j_* and *C_j+1_* are two adjacent clusters, *N_1_* and *N_2_* are the number of RPs in *C_j_* and *C_j+1_*, respectively;6.  *temp* = [];7.  **for**
*k* = 1 to *N_1_*
**do**8.   *temp* = find(*C_j+_*_1_.*x* == *C_j,k_*.*x* & *C_j+_*_1_.*y* == *C_j,k_*.*y*), *C_j,k_* denotes the *k*th RP in *C_j_*, *C_j,k_*.*x* denotes the *x* coordinate of the *k*th RP in *C_j_*, *C_j+_*_1_.*x*, *C_j+_*_1_.*y* and *C_j,k_*.*y* are in similar way, *temp* denotes the indexes of RPs in *C_j+_*_1_ with same coordinates of the *k*th RP in *C_j_*;9.   *idx1* = [*idx1*; *temp*], *idx1* denotes the indexes of selected RPs in *C_j+_*_1_;10.  **end for**11.  *temp* = [];12.  **for**
*l* = 1 to *N_2_*
**do**13.   *temp* = find(*C_j_*.*x* == *C*_*j+1,l*_.*x* & *C_j_*.*y* == *C_j+_*_1*,l*_.*y*);14.   *idx2* = [*idx2*; *temp*], *idx2* denotes the indexes of selected RPs in *C_j_*;15.  **end for**16.  *N_tr1_*= Num(*idx1*), *C_tr1_* = *C_j+_*_1*,idx*1_, *N_tr_*_2_ = Num(*idx2*), *C_tr_*_2_ = *C_j,idx_*_2_, *C_tr_*_1_ denotes the corresponding RPs of *idx1* in *C_j+_*_1_, *N_tr_*_1_ denotes the number of *C_tr_*_1_, *N_tr_*_2_ and *C_tr_*_2_ are in similar way;17.  *temp* = [];18.  **for**
*m* = 1 to *N_tr_*_1_
**do**19.   *temp* = find(dist(*C_tr_*_1*,m*_, *C_j+_*_1_) ≤ *d_int_*), dist(*C_tr_*_1*,m*_, *C_j+_*_1_) denotes distances between the *m*th RP in *C_tr_*_1_ and all RPs in *C_j+_*_1_, *temp* denotes the indexes of RPs *d_int_* away from the *m*th RP in *C_tr_*_1_ in *C_j+_*_1_;20.   *id1* = [*id1*; *temp*], *id1* denotes the indexes of RPs within the range of *d_int_* in *C_j+_*_1_;21.  **end for**22.  *C_adj1_*= *C_j+1,id1_*, denotes that RPs within the range of *d_int_* in *C_j+1_* for *C_tr1_* are selected;23.  *temp* = [];24.  **for**
*n* = 1 to *N_tr2_*
**do**25.   *temp* = find(dist(*C_tr_*_2,*n*_ , *C_j_*) ≤ *d_int_*)26.   *id2* = [*id2*; *temp*];27.  **end for**28.  *C_adj_*_2_ = *C_j,id1_*;29.  *C_j_* = unique(*C_j_* + *C_tr_*_1_ + *C_adj_*_1_), *C_j+_*_1_ = unique(*C_j+_*_1_ + *C_tr_*_2_ + *C_adj_*_2_);30.  update cluster centers of *C_j_* and *C_j+_*_1_;31. **end for**

In Algorithm 1, Num() is a counting function, find() is the function of finding RPs which meets the specified conditions and responding the corresponding indexes, dist() is the function of distance between two RPs, unique() is the function returns the same values but with no repetitions. Line 7 to Line 9 are used for finding RPs in one cluster with same coordinates of RPs in the other cluster. Until Line 16, the algorithm can find all RPs with same coordinates in both two adjacent clusters, i.e., the combination of *C_tr_*_1_ and *C_tr_*_2_. Then, RPs *d_int_* away from transition region in both two adjacent clusters can be selected. Subsequently, both two adjacent clusters are updated by Line 29. The unique() function can ensure that there are no repetitive RPs in cluster. Finally, the cluster center can be updated, and when the loop is terminated, the output of the algorithm is the combination of all updated clusters, i.e., IOFPD. 

### 2.5. The Adaptive Weighted K-Nearest Neighbor Localization

We adopt the common cluster matching method to select proper clusters by comparing the similarity of the online RSS readings and each cluster center. It is inevitable that the common cluster matching method has a low accuracy, because of the dynamic and sophisticated indoor environment. As cluster matching is not the emphasis of this work, we pay more attention to the adaptive weighted K-nearest neighbor localization algorithm.

After cluster matching, the selected cluster is utilized for further matching with the real-time RSS readings. We put forward a novel AWKNN algorithm for estimating positions based on KNN, APC and adaptive weighted algorithm. KNN is one of the most well-known machine learning algorithms, and it is widely used for estimating position in fingerprint localization methods. In our proposed AWKNN algorithm, we first choose K (K is larger than 5) initial RPs with K top smallest signal-domain distances between RPs and the online RSS readings by KNN algorithm. Then, the K initial RPs are clustered by APC for dividing into several sub-clusters. Next, the most probable sub-cluster is reserved based on its amount of RPs and the signal-domain distance between sub-cluster center and the online RSS readings. The following is estimating user’s position by the inverse distance weighted algorithm based on the reserved sub-cluster. In this subsection, KNN algorithm is not described in detail because of our adoption with no modification but the calculation of the average signal-domain distance.

K initial RPs with signal-domain distances can be obtained by the first-level KNN algorithm. They are automatically divided into several sub-clusters with high-quality clustering results by APC. With the help of APC, the K initial RPs can be divided into several sub-clusters. The following task is to estimate the user’s position. In a word, the basic idea of the proposed AWKNN algorithm is to select several concentrated RPs for weighted average coordinates calculation based on their positions and similarities (signal-domain distances). By this means, the calculated results are more convergent avoiding large positioning error. The Algorithm 2 is described in detail as follows.

**Algorithm 2:** The proposed AWKNN localization algorithm1. **Input:** the online RSS readings *r*, selected cluster (*C_s_*) after cluster matching, K, the number of RPs in selected cluster (*n*)2. **Output:** weighted average coordinates3. **if**
*n* < 10 **then**4.  K = *n*5. **else**6.  K = 107. **end if**8. **for**
*i* = 0 to *n*
**do**9.  calculate signal-domain distances (*d_sig_*) between each RP in *C_s_* and *r* by Equation (3)10. **end for**11. obtain K initial RPs with K top smallest *d_sig_*12. divide these K initial RPs into several sub-clusters by affinity propagation clustering, the number of sub-clusters is denoted as *N_c_*13. **if**
*N_c_* == 1 **then**14.  calculate coordinates with K initial RPs by Equation (12)15. **else if**
*N_c_* ≥ K-3 **then**16.  calculate coordinates with 3 RPs that have top smallest *d_sig_* by Equation (12)17. **else**18.  obtain the number of RPs in each sub-cluster, denoted as *N_RP_*19.  sort(*N_RP_*) in descending order20.  select 2 sub-clusters (*C_sub,_*_1_ and *C_sub,_*_2_) with 2 top biggest *N_RP_*, that is, *N_RP_*_1_ and *N_RP_*_2_, and the *d_sig_* of sub-cluster centers are *d*_1_ and *d*_2_, respectively^1^21.  *N_diff_* = *N_RP_*_1_ − *N_RP_*_2_22.  **if**
*N_diff_* ≥ 3 **then**23.    calculate coordinates with the sub-cluster *C_sub_*_,1_, of which the number is *N_RP1_*24.  **else**25.   **if**
*d*_1_ ≦ *d*_2_
**then**26.    calculate coordinates with the *C_sub,_*_1_ by Equation (12)27.   **else**28.    calculate coordinates with the *C_sub_*_,2_ by Equation (12)29.   **end if**30.  **end if**31. **end if**

Unlike the K-means or FCM clustering algorithm, cluster centers are certain sample points themselves in affinity propagation algorithm. In the above algorithm, the signal-domain distance between RP and the online RSS readings has been calculated at line 9. Therefore, the signal-domain distances between sub-cluster centers and the online RSS readings are no need to re-compute. In addition, the signal-domain distances are denoted as *d*_1_ and *d*_2_ for convenience, respectively.

We suppose that there are *m* remaining RPs in the aim sub-cluster, the user’s position can be estimated by Equation (12), where *x* and *y* are coordinate values, and *d* is the corresponding signal-domain distance:

(12) $$\{\begin{array}{c} \overset{\wedge }{x}=\sum_{i=1}^{m}\left(x_{i}\times \frac{1}{d_{i}}\right)/\sum_{i=1}^{m}\frac{1}{d_{i}}\\ \overset{\wedge }{y}=\sum_{i=1}^{m}\left(y_{i}\times \frac{1}{d_{i}}\right)/\sum_{i=1}^{m}\frac{1}{d_{i}} \end{array}\;$$

**Figure 3:**
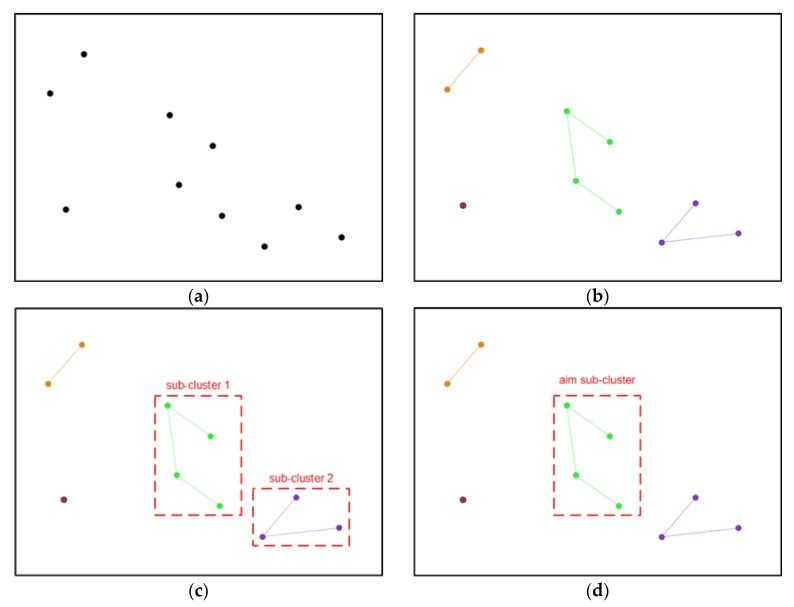
The illustration of the proposed AWKNN localization algorithm, (**a**) get 10 initial RPs from the selected cluster by KNN; (**b**) 4 sub-clusters are divided by APC and illustrated in different colors; (**c**) select 2 sub-clusters with top number of points; (**d**) obtain the aim sub-cluster by the comparison of signal-domain distance between sub-cluster center and the online RSS readings.

Figure 3 shows an illustration of the proposed AWKNN localization algorithm. In Figure 3a, 10 initial RPs are first selected based on signal-domain distances by KNN algorithm. The 10 points are in black. Then, they are automatically divided into four sub-clusters by APC algorithm. All sub-clusters are described in 4 different colors, as is shown in Figure 3b. The number of sub-clusters are 1, 2, 3 and 4, respectively. Based on the above algorithm description from line 13 to line 20, we can get two sub-clusters with the two topmost RPs. These two sub-clusters are selected in red dotted box. Seen from Figure 3c, they are sub-cluster 1 and sub-cluster 2, respectively. Then, the aim sub-cluster is found based on signal-domain distance between the corresponding center and the online RSS readings, as described at line 26. The aim sub-cluster for next estimating position is illustrated in red dotted box of Figure 3d.

## 3. Experiment and Results

This section shows the experimental testbed and evaluates the performances of different positioning approaches.

### 3.1. Experimental Setup

**Figure 4:**
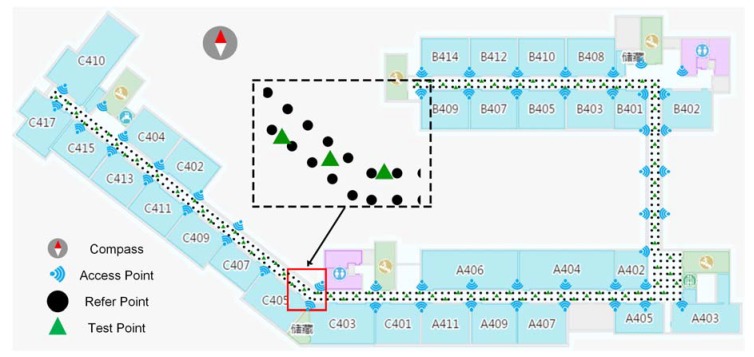
The layout of experimental testbed, the solid dot is refer point, the solid triangle is test point, and the signal mark refers to Wi-Fi access point.

The experiment is conducted on the 4th floor of an office building, occupied by School of Environment Science and Spatial Informatics, China University of Mining and Technology (CUMT, Xuzhou, China). The area of the testbed is almost 3200 square meters. The layout is shown in Figure 4. Fifty five TP-Link 2.4 GHz wireless access points (APs) are pre-installed at about 3 m above the floor. The solid dots refer to the reference points with a total of 374. The sampling period is 60 s in each direction, and the sampling frequency is 1 Hz. The distance between two adjacent RPs is 1.2 m. Due to the RSS collections in four difference orientations, the number of RPs is 1516 in the fingerprint database. On average, the mobile device could receive at least 8 APs’ Wi-Fi signals at each location. The RSS data collection was conducted by eight postgraduates with different smart phones, such as Mi. 6, Mi. 5X, Huawei Mate8, and Samsung Galaxy S7.

To test the performance of the proposed positioning approach, 86 test points (TPs), described as green solid triangle, lie evenly in the experiment area, as shown in Figure 4. The operation of RSS measurements at each TP is the same as the above operation, but the sampling period is 10 s in each direction. There are 3440 sets of testing data in total.

### 3.2. Clustering Results

This subsection successively displays the clustering results of CFPD and IOFPD by APC algorithm. The CFPD has 374 RPs, which takes average of the above RSS measurements at the same location in different orientations, but the IOFPD has more than 1600 RPs because of the adjusted transition region of adjacent clusters. 

**Figure 5:**
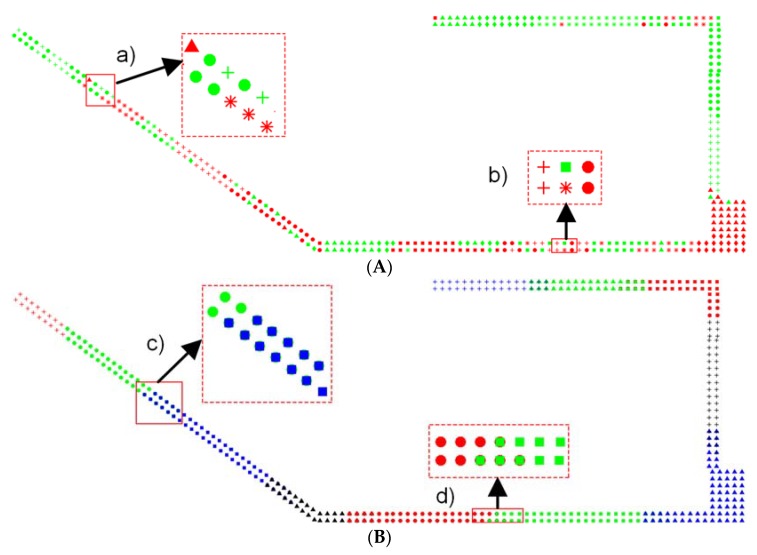
The clustering results of CFPD and IOFPD, where point marks mean different cluster results, (**A**) the clustering results of CFPD with several inaccurate cluster division; (**B**) the clustering results of IOFPD with adjusted transition regions.

The clustering for CFPD is conducted by taking the signal-domain distance as the clustering feature with 12 clusters, while there are 11 clusters for IOFPD based on the fusion of signal-domain distance and position-domain distance. The clustering results of IOFPD can exactly reflect the layout of experimental testbed. As is shown in Figure 5, different colors and shapes denote different clusters. Figure 5A shows the clustering for CFPD based on the signal-domain distance with several inaccurate cluster division in both (a) and (b) dash boxes, where have several different clusters; (b) shows the clustering IOFPD based on the hybrid distance with a good clustering performance, and adjusted transition regions in both (c) and (d) dash box.

### 3.3. Positioning Results

This subsection mainly describes the positioning results. The existing WKNN algorithm is introduced for comparing with our proposed AWKNN algorithm. And the maximum error (ME), error mean (EM), and root mean square error (RMSE) are utilized for evaluating the performances of different positioning methods.

Both the values of EM and RMSE are minimum based on the CFPD by WKNN algorithm when K is 6. And the best positioning performance is obtained for the IOFPD_WKNN method when K is 9. While the methods based on both the CFPD and IOPFD by AWKNN algorithm can adaptively select different K refer points for estimating positions.

**Figure 6:**
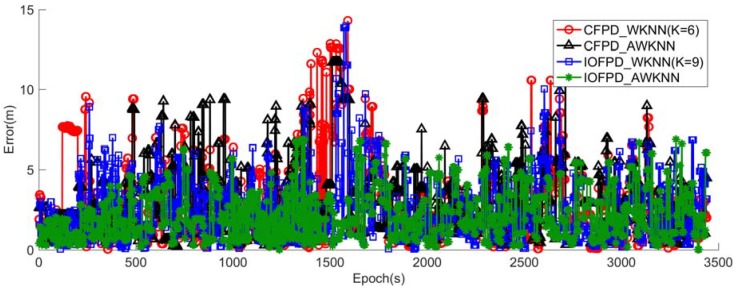
The positioning errors of the CFPD_WKNN method, the CFPD_AWKNN method, the IOFPD_WKNN method and the IOFPD_AWKNN method, where the CFPD_WKNN and IOFPD_WKNN method get the best positioning performance when K are 6 and 9, respectively.

Figure 6 illustrates the epoch-epoch positioning errors of the CFPD_WKNN method (red curve with circles), the IOFPD_WKNN method (black curve with triangles), the CFPD_AWKNN method (blue curve with squares) and IOFPD_AWKNN method (dark green curve with stars) by using the same testing data. The dark green curve with stars fluctuates slightly, while the red curve with circles fluctuates obviously. In other words, the IOFPD_AWKNN method has less positioning errors, while the CFPD_WKNN method has larger positioning errors. It is also obvious that the localization performance of CFPD-based methods (CFPD_WKNN and CFPD_AWKNN) is poorer than the IOFPD-based methods (IOFPD_WKNN and IOFPD_AWKNN).

As shown in Table 2, the IOFPD_AWKNN method is implemented with the maximal error of 6.8 m, the mean error of 2.2 m, the root mean square error of 1.5 m, while the other three positioning methods have larger errors than the proposed method. Comparing with the CFPD_WKNN method, it achieves an ME improvement of 7.427 m (51.9%), an EM improvement of 0.57 m (20.5%), and an RMSE improvement of 0.918 m (37.8%) at least, because the CFPD_WKNN method gets the best positioning performance when K is 6. Comparing with the CFPD_AWKNN method, it achieves an ME improvement of 4.841 m (41.3%), an EM improvement of 0.63 m (22.1%), and an RMSE improvement of 0.808 m (34.9%). Comparing with the IOFPD_WKNN method, it achieves an ME improvement of 7.089 m (50.7%), an EM improvement of 0.263 m (10.6%), and an RMSE improvement of 0.437 m (22.4%). Similarly, the CFPD-based methods have a lager ME than the IOFPD-based methods. The EM of the CFPD-based methods decrease at least 0.46 m.

**Figure 7:**
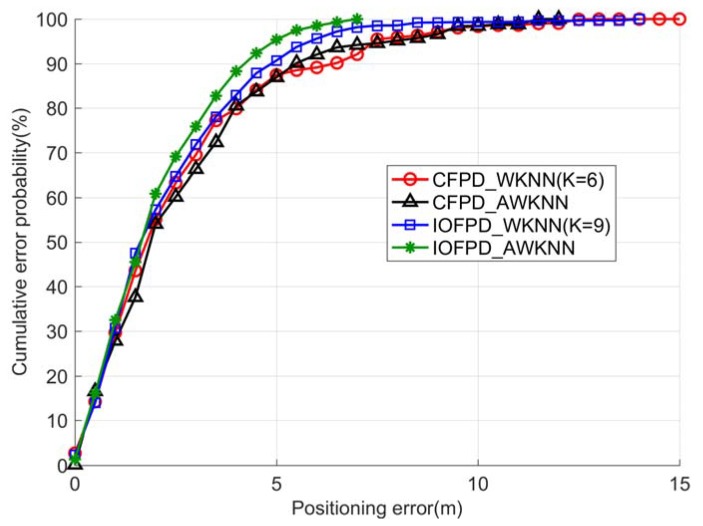
The cumulative probability distributions of the positioning errors related to the CFPD_WKNN method, the CFPD_AWKNN method, the IOFPD_WKNN method and the IOFPD_AWKNN method, where the CFPD_WKNN and IOFPD_WKNN method get the best positioning performance when K are 6 and 9, respectively.

Figure 7 shows the cumulative probability distributions of the four positioning methods. The four positioning methods have consistent performance when the positioning error is less than 1 m. But the IOFPD_AWKNN method performs better than the other three methods when the positioning error is larger than 2 m.

It can be seen from the Figure 6, Table 2 and Figure 7 that the proposed method (IOFPD_AWKNN) has a better positioning performance than the other three methods (CFPD_WKNN, CFPD_AWKNN and IOFPD_WKNN). The employed IOFPD has a better localization performance than the fingerprint database without considering users’ orientations. The proposed method achieves the mean error of 2.2 m, the root mean square error of 1.5 m in the almost 3200 square meters testbed.

## 4. Discussion

Through comparing the positioning results of the above four methods, we can find that the proposed method (IOFPD_AWKNN) has a better positioning performance. It seems that the proposed IOFPD_AWKNN method can constrain large localization errors and make both the mean error and root square mean error small.

The IOFPD is rather useful to improve positioning accuracy. No matter which estimation algorithms are adopted, the positioning performance of IOFPD-based methods outperform CFPD-based methods. IOFPD-based methods has small maximum error, mean error and root square mean error. In addition, the proposed AWKNN algorithm also has the benefit of improving positioning performance. For example, the existing CFPD_WKNN method has a larger maximum error and root square mean error than CFPD_AWKNN method, as well as IOFPD_WKNN and IOFPD_AWKNN methods. It is obvious that no matter which fingerprint database are selected, the proposed AWKNN algorithm leads to small maximum error and root square mean error. However, the proposed AWKNN algorithm plays an unconsidered role based on CFPD, because of consistent cdf curves and common positioning accuracy of CFPD_WKNN and CFPD_AWKNN methods. It is understandable and acceptable because AWKNN algorithm needs more RPs to select concentrated ones for final location estimation. In this way, more RPs in CFPD means a large wide coverage. These scattered RPs are divided into so much sub-clusters by affinity propagation clustering algorithm. If the number of sub-clusters is bigger than K-3, the proposed AWKNN algorithm is equivalent to WKNN algorithm, besides selecting three RPs with top smallest signal-domain distances for estimating position. In addition, the IOPFD and AWKNN cooperate with each other, and the IOFPD_AWKNN has a best positioning performance with the mean error of 2.2 m, the root mean square error of 1.5 m.

Although the IOFPD makes a contribution to improve the localization accuracy of the proposed AWKNN algorithm, the IOFPD construction is time-consuming and labor-sensitive. It is a big challenge for all fingerprint-based positioning methods. Therefore, several techniques should be introduced for the further research to reduce the workload of fingerprint acquisition, such as sensor-based crowdsourcing, Gaussian process, the path loss model-based and adaptive update fingerprint technologies, etc.

## 5. Conclusions

This work proposes an adaptive weighted K-nearest neighbor fingerprinting method based on omnidirectional fingerprint database and twice affinity propagation clustering. The most innovative is taking position-domain distribution of RPs into account for estimating user’s position by affinity propagation clustering on the online stage. Meanwhile, to avoid mismatching caused by transition region between two adjacent clusters, we propose the adjusting cluster algorithm based on transition region. The experimental results demonstrate that the proposed IOFPD_AWKNN positioning method outperforms the traditional fingerprinting positioning methods.

## Figures and Tables

**Table 1:** The omnidirectional fingerprint database.

id	pid	*x* (m)	*Y* (m)	Orientation (°)	MAC	RSS(dBm)
1	1	0.0	0.0	23.5	6c:e8:73:91:96:d0	−68
2	1	0.0	0.0	23.5	6c:e8:73:91:96:a1	−47
15	1	0.0	0.0	23.5	6c:e8:73:90:97:d5	−63
28	2	0.0	0.0	115	6c:e8:73:91:96:d0	−75

**Table 2:** The positioning error statistics of the CFPD_WKNN method, CFPD_AWKNN method, IOFPD_WKNN method and IOFPD_AWKNN method (Unit: m).

Methods	ME	EM	RMSE
CFPD_WKNN (K = 6)	14.304	2.785	2.427
CFPD_AWKNN	11.718	2.845	2.317
IOFPD_WKNN (K = 9)	13.966	2.478	1.946
IOFPD_AWKNN	6.877	2.215	1.509
